# From Architecture to Outcomes: Mapping the Landscape of Digital Twins for Personalized Diabetes Care—A Scoping Review

**DOI:** 10.3390/jpm15110504

**Published:** 2025-10-23

**Authors:** Danilo Andrés Cáceres-Gutiérrez, Diana Marcela Bonilla-Bonilla, Yamil Liscano, Jhony Alejandro Díaz Vallejo

**Affiliations:** 1 Specialization in Internal Medicine, Department of Health, Universidad Santiago de Cali, Cali 760035, Colombia; danilo.caceres00@usc.edu.co (D.A.C.-G.); diana.bonilla01@usc.edu.co (D.M.B.-B.); 2 Department of Research and Education, Clínica de Occidente S.A., Cali 760046, Colombia; 3 Grupo de Investigación en Salud Integral (GISI), Departamento Facultad de Salud, Universidad Santiago de Cali, Cali 760035, Colombia; 4 Research Group on Nutrition, Metabolism and Food Safety, Basic Health Sciences Department, University of Caldas, Manizales 170004, Colombia; alejandrodiazval@gmail.com

**Keywords:** digital twins, diabetes, predictive modeling, personalized care

## Abstract

**Background/Objectives**: Digital twins are emerging as a transformative technology in diabetes management, promising a shift from standardized protocols to highly personalized care. This scoping review aims to systematically map the current landscape of digital twin applications in diabetes, synthesizing evidence on their implementation architectures, analytical models, performance metrics, and clinical integration strategies to identify key trends and critical gaps. **Methods**: A systematic search was conducted across five electronic databases in accordance with PRISMA-ScR guidelines to identify empirical studies on digital twins for diabetes. Data from the selected articles were extracted to analyze bibliographic characteristics, population data, technological components, performance outcomes, and integration levels. A narrative synthesis was performed to map the evidence. **Results**: Seventeen studies were included, revealing a rapid increase in publications since 2022, with a notable concentration of research in India. The technological architecture shows a convergence toward machine learning models (e.g., LSTM) powered by data from IoT devices and wearables. Certain interventional studies have reported significant clinical impacts, including HbA1c reductions of up to 1.9% and T2DM remission rates as high as 76.5% in one trial. However, major implementation barriers were identified, including fragmented interoperability standards and low rates of full integration into clinical workflows (35.3%). **Conclusions**: Digital twins are emerging as powerful tools that show the potential to drive significant clinical outcomes in diabetes care. However, to translate this promise into widespread practice, future efforts must focus on overcoming the critical challenges of standardized interoperability and deep clinical integration. Rigorous, independently validated, long-term trials in diverse populations are essential to confirm these promising findings.

## 1. Introduction

The management of diabetes mellitus is undergoing a paradigm shift, moving away from standardized protocols toward highly personalized therapeutic strategies. At the forefront of this transformation is the digital twin, a sophisticated virtual representation of an individual patient, which promises to revolutionize diabetes care by integrating real-time, multi-stream data to simulate and predict physiological responses [1,2,3]. By creating a dynamic model of each patient, digital twins offer the potential to move beyond reactive adjustments and enable proactive, predictive, and truly personalized medicine [4].

The field of digital twins for diabetes is experiencing rapid growth, as evidenced by the surge in publications in recent years. This acceleration has produced a diverse and complex body of evidence, spanning from foundational conceptual frameworks to advanced clinical trials reporting promising improvements in glycemic control and, in some cases, disease remission [2,4,5]. However, this rapid development has also led to a fragmented landscape of technological architectures, validation methodologies, and clinical integration strategies. Without a clear synthesis of the current state of the art, it is challenging for researchers, clinicians, and policymakers to identify robust trends, critical knowledge gaps, and the most promising pathways for translating these powerful tools into routine clinical practice [6,7].

The core mission of personalized medicine is to harness technological innovation to deliver tangible clinical benefits tailored to the individual. Digital twins epitomize this goal by offering a mechanism to decode a patient’s unique metabolic profile and optimize their treatment in real-time. Understanding the current implementation status, performance benchmarks, and integration challenges of these systems is therefore essential for advancing the field of precision diabetology [7,8,9].

Therefore, this scoping review aims to systematically map the current landscape of digital twin applications in diabetes management. We synthesize the available evidence on implementation architectures, analytical models, performance metrics, and clinical integration strategies. By providing a comprehensive overview of this rapidly evolving field, we seek to identify key trends and critical gaps, thereby informing future research and guiding the evidence-based implementation of digital twins to advance personalized diabetes care and improve patient outcomes.

## 2. Methods

### 2.1. Methodological Framework and Protocol

This scoping review was conducted in accordance with the Joanna Briggs Institute (JBI) guidelines for scoping reviews and the PRISMA-SCR (Preferred Reporting Items for Systematic Reviews and Meta-Analyses Extension for Scoping Reviews) framework [10,11]. The protocol was designed to comprehensively explore the use of digital twins in diabetes monitoring, treatment, and personalized management, providing a systematic mapping of the available evidence in this rapidly evolving field.

### 2.2. Key Definitions

To ensure conceptual consistency throughout the review process, the following operational definitions were established. Digital twins in diabetes were defined as dynamic virtual replicas of diabetic patients that integrate real-time data from multiple sources including wearable devices, implantable sensors, and electronic health records to simulate, predict, and optimize personalized diabetes management. Traditional diabetes monitoring encompassed conventional surveillance methods including periodic blood glucose measurements, HbA1c testing, and scheduled clinical assessments. Digital personalized medicine referred to therapeutic approaches utilizing computational algorithms and individual patient data to customize treatments and predictions in real-time. Complication prediction was characterized as the capacity of digital systems to anticipate adverse events such as hypoglycemic or hyperglycemic episodes before clinical manifestation [1,2,4]. Type 2 Diabetes Remission was defined according to the 2021 ADA consensus criteria as achieving and maintaining an HbA1c level below 6.5% for at least three months without the use of glucose-lowering pharmacotherapy.

### 2.3. Research Question and PCC Framework

The structured PCC question framework guided this review where Population comprised human patients diagnosed with diabetes mellitus type 1 or type 2 of any age and geographical context, Concept focused on the implementation and effectiveness of digital twins for real-time monitoring, predictive modeling, and diabetes treatment personalization, and Context encompassed empirical studies published in clinical, research, or community settings evaluating digital twin applications in diabetes management.

The refined research question was formulated as follows: How are digital twins implemented in comprehensive diabetes management, what analytical methods do they employ, what are their performance metrics in predicting complications and personalizing treatment, and how do they integrate with traditional diabetes care systems?

### 2.4. Search Strategy

A systematic and comprehensive search strategy was designed and executed on 25 May 2025, across 5 electronic databases including PubMed, Scopus, Web of Science, ScienceDirect, and Google Scholar. The search strategy was constructed using key terms related to the components of the PCC question framework, with no language or date range filters applied during the database search to maximize evidence retrieval.

Search Algorithm:

(“Digital Twin*” OR “Digital Twins” OR “Virtual Patient*” OR “Patient Digital Model*” OR “Computational Patient Model*”) AND (“Diabetes Mellitus” OR “Diabetes” OR “Diabetic*” OR “Type 1 Diabetes” OR “Type 2 Diabetes” OR “T1DM” OR “T2DM” OR “Insulin Dependent” OR “Non-insulin Dependent”) AND (“Real-time monitoring” OR “Continuous monitoring” OR “Predictive model*” OR “Personalized treatment” OR “Precision medicine” OR “Individualized therapy” OR “CGM” OR “Continuous glucose monitor*” OR “Wearable device*” OR “IoT healthcare” OR “Machine learning” OR “Artificial intelligence” OR “AI healthcare”) NOT (“Review” OR “Systematic review” OR “Meta-analysis” OR “Commentary” OR “Editorial”)

This comprehensive algorithm was adapted for each database platform to accommodate specific indexing systems and search functionalities, ensuring optimal retrieval of relevant literature across all platforms.

### 2.5. Study Selection Process

Following the search execution, all identified records were exported to Zotero version 6.0 for citation management and duplicate removal. The remaining records were subsequently uploaded to Rayyan AI (https://www.rayyan.ai/ (accessed on 25 May 2025)) collaborative screening platform accessed on 25 May 2025, to facilitate the systematic review process. Two independent reviewers (D.A.C.G. and Y.L.) conducted initial screening of titles and abstracts to determine study eligibility based on the criteria outlined in Table 1. In accordance with our search strategy, articles identified as reviews, commentaries, or editorials during this stage were excluded. Articles meeting initial screening criteria underwent full-text evaluation by the same reviewers to assess final inclusion. Disagreements between reviewers were resolved through consensus discussion or consultation with a third reviewer (D.M.B.B.) when necessary. The complete study selection process was documented using a PRISMA flow diagram (https://estech.shinyapps.io/prisma_flowdiagram/ (accessed on 25 May 2025 ) [12] to ensure transparency and reproducibility.

### 2.6. Data Extraction

A structured data extraction form was developed to capture a comprehensive range of variables relevant to the study objectives. The extraction process was conducted systematically by two independent reviewers to ensure accuracy and completeness of data collection. The extracted variables were organized into five main categories as detailed in Table 2.

### 2.7. Statistical Analysis and Data Synthesis

A descriptive and narrative synthesis approach was used to map the evidence. Consistent with the PRISMA-ScR guidelines for scoping reviews, a quantitative meta-analysis was not performed, as the primary goal was to map the scope and nature of the evidence, not to produce a pooled statistical estimate of effect. The significant heterogeneity observed across study designs, populations, intervention specifics, and reported outcomes further supports the appropriateness of a narrative synthesis. Data were analyzed using frequency counts for categorical variables (e.g., study design, geographical distribution, interoperability standards, system integration levels) and descriptive statistics (e.g., medians, ranges) for continuous variables (e.g., sample size, performance metrics). This synthesis allowed for the identification of key trends, such as publication patterns over time and the evolution of technological approaches. All data visualizations, including charts and diagrams summarizing the findings, were generated using Python (version 3.9.7 accessed on 25 May 2025) with the Matplotlib (Version 3.9.1, The Matplotlib Development Team/NumFOCUS, Austin, TX, USA) and Seaborn (Version 0.13.2, Michael Waskom and the Seaborn development team, New York, NY, USA) libraries to ensure a comprehensive and clear representation of the results.

### 2.8. Methodological Considerations

In line with the JBI guidelines for scoping reviews, a formal quality assessment of the included studies was not performed. However, to contextualize the findings and understand the maturity of the evidence base, key methodological characteristics were systematically documented. This included noting the study design, validation methods (e.g., internal vs. external), sample size, and the completeness of outcome reporting. Potential sources of bias and limitations related to the generalizability of the findings were considered during the narrative synthesis to inform the interpretation of the results, identify knowledge gaps, and formulate recommendations for future research.

## 3. Results

### 3.1. Study Selection

**Figure 1:**
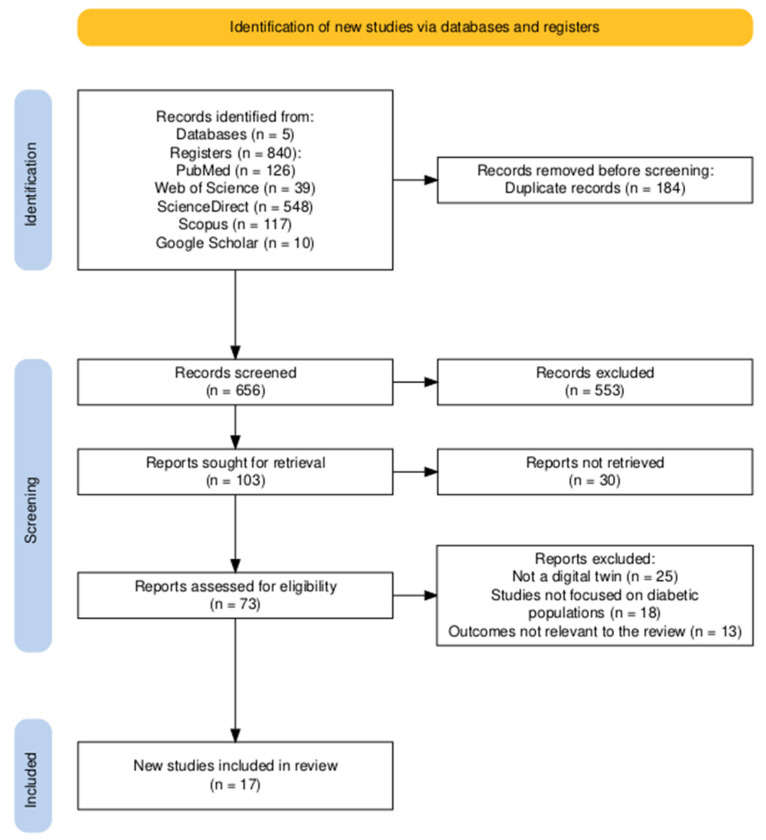
PRISMA flow diagram illustrating the search and screening process. The level of agreement between reviewers during study selection was considered moderate to substantial, with Cohen’s Kappa coefficients of 0.76 for title and abstract screening and 0.95 for full-text eligibility assessment. These values reflect a generally consistent, though not perfect, alignment between reviewers throughout the selection phases.

This PRISMA flow diagram (Figure 1) illustrates a methodologically rigorous and transparent study selection process for a systematic review. The procedure began with an identification phase where a broad search across five databases yielded 840 records, which were narrowed down to 656 unique articles after the removal of 184 duplicates. During the screening phase, a substantial number of records, 553, were excluded based on their title and abstract, after which 103 reports were sought for full-text review. Of these, 30 reports could not be retrieved for a final eligibility assessment. The primary reasons for non-retrieval were institutional paywall restrictions for certain journals and, in a smaller number of cases, broken or defunct repository links for older conference proceedings. The remaining 73 articles underwent a detailed eligibility assessment, leading to the exclusion of 56 for clearly specified reasons: 25 were not about a digital twin, 18 did not focus on diabetic populations, and 13 had irrelevant outcomes. This meticulous process resulted in a final set of 17 studies included in the review. The strength of this methodology is significantly enhanced by the reported inter-rater reliability, with Cohen’s Kappa coefficients of 0.76 for abstract screening and 0.95 for full-text assessment, indicating substantial to almost perfect agreement. Overall, the diagram and associated data demonstrate a robust, reproducible, and high-quality selection process that minimizes bias and adheres to established scientific standards.

### 3.2. Bibliographic and Methodological Characteristics

**Figure 2:**
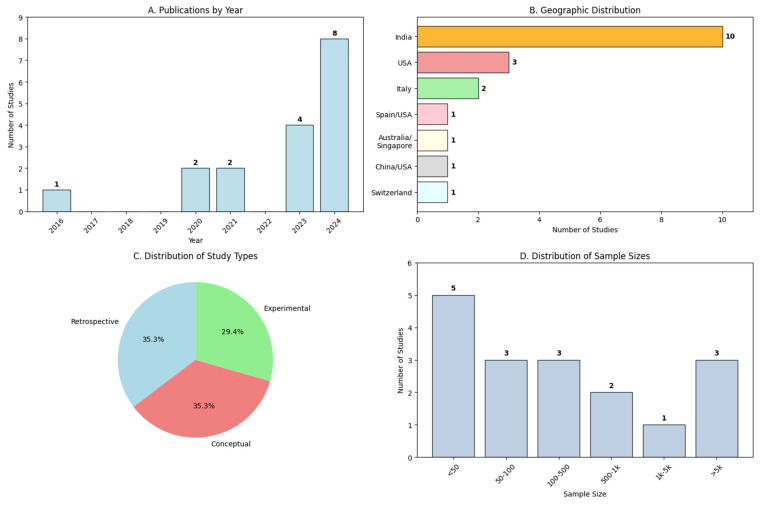
Overview of digital twin literature in diabetes. (**A**) Shows the number of publications by year, with a peak in 2024. (**B**) Depicts the geographic distribution of the studies, identifying India as the leading contributor. (**C**) Displays the proportional distribution of study types, classified as retrospective (35.3%), conceptual (35.3%), and experimental (29.4%). (**D**) Presents the distribution of sample sizes across studies, with most studies having fewer than 50 participants. Note: This figure was generated using Python.

The analysis of the 17 included studies reveals clear temporal evolution in digital twin research applied to diabetes (Figure 2A). Research began in 2016 with a pioneering study, followed by a consolidation period with 2 studies in 2020 and 2 in 2021. The field experienced significant acceleration from 2023 onwards, with 4 studies published that year, reaching its peak in 2024 with 8 publications, representing 47.1% of all scientific production in this domain.

The geographical distribution of research shows a notable concentration in India, contributing 10 of the 17 studies (58.8%), primarily from the research group led by Shamanna et al. at the Centre for Chronic Disease Control (see Table 3). This geographical dominance reflects both the high prevalence of diabetes in the region and the development of advanced technological infrastructures for digital health. The remaining 7 studies (41.2%) are distributed among the United States (2 studies), Europe (3 studies), and other Asian countries (2 studies).

From a methodological perspective, studies are classified into three main categories (Figure 2C): retrospective studies representing 35.3% (6 studies), conceptual/theoretical studies also with 35.3% (6 studies), and experimental/prospective studies with 29.4% (5 studies). This balanced distribution indicates growing maturity in the field, with a balance between conceptual development, retrospective validation, and prospective experimentation.

### 3.3. Population Characteristics

Analysis of population characteristics reveals the diversity of clinical contexts addressed by digital twin systems in diabetes (Table 4). The majority of studies focused on Type 2 Diabetes Mellitus, reflecting the higher prevalence of this condition and the complexity of its management. Four studies specifically addressed Type 1 Diabetes Mellitus, while one study did not specify the diabetes type as it was a conceptual framework.

Sample sizes across studies are highly heterogeneous, ranging from small feasibility trials with 5 participants to large retrospective analyses involving over 7000 individuals. This variability highlights the mix of exploratory research and large-scale validation efforts within the field. The demographic data generally pertains to adult populations, with ages ranging from pediatric cohorts (8–14 years) to older adult populations with means approaching 61 years. Gender distribution, where reported, shows relatively balanced representation, with female participation ranging from 16.3% to 73% across studies.

Analysis of baseline glycemic control reveals significant variability, often reflecting the study’s purpose. Interventional T2DM trials frequently enrolled participants with poor glycemic control, with mean baseline HbA1c values ranging from 8.1% to 9.0%. In contrast, large-scale modeling studies that included healthier individuals reported much lower mean values, such as 5.51%. Studies focused on T1DM also showed varied baselines, for instance, 7.41% in one of the included cohorts.

The frequent reporting of common comorbidities such as hypertension, chronic kidney disease, metabolic dysfunction-associated fatty liver disease, and cardiovascular conditions indicates that these digital twin models are being developed to address the complex, real-world clinical profiles of individuals with diabetes. This complexity is further illuminated by the specific eligibility criteria used. Notably, many interventional studies required adequate hepatic and renal function for inclusion and often excluded patients with recent cardiovascular events, reflecting the need for stable physiological conditions to validate digital twin predictions. Furthermore, some of the most successful T2DM remission trials specifically enrolled participants with a shorter disease duration (e.g., <8 years), a factor that may influence the reported high efficacy rates.

### 3.4. Digital Twin Implementation and Technology

**Figure 3:**
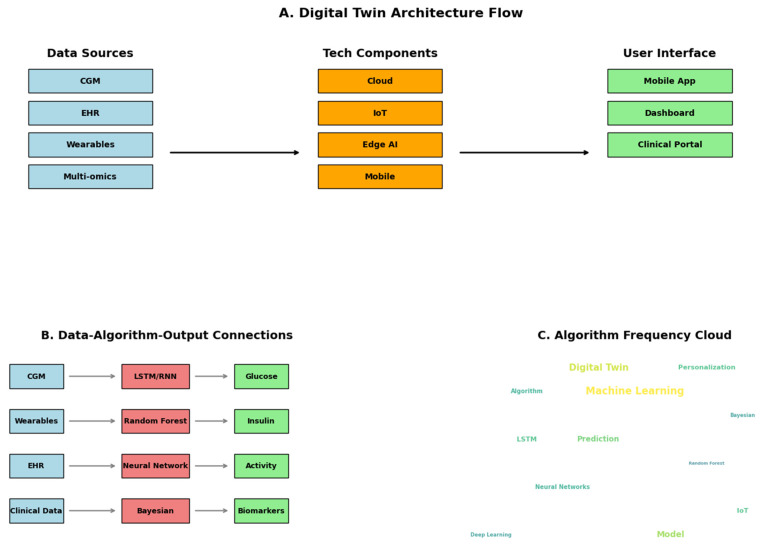
Technological architecture and algorithmic models in digital twin implementation. (**A**) Displays a flowchart of the general architecture of digital twins in diabetes, showing the progression from data sources (e.g., CGM, EHR, wearables, multi-omics) to technological components (e.g., cloud, IoT, Edge AI), and finally to user-facing interfaces such as mobile apps and clinical dashboards. (**B**) Illustrates typical data–algorithm–output connections, mapping data types to machine learning models (e.g., LSTM, Random Forest, Bayesian) and their respective outputs (e.g., glucose, insulin, biomarkers). (**C**) Presents a word cloud of frequently used algorithms and terms in the analyzed literature, where word size reflects relative frequency. Abbreviations: CGM = continuous glucose monitoring; EHR = electronic health record; IoT = Internet of Things; AI = Artificial Intelligence; LSTM = Long Short-Term Memory; RNN = Recurrent Neural Network. Note: This figure was generated using Python.

The analysis of digital twin implementations, summarized in Table 5 and visually depicted in Figure 3, reveals a consistent architectural framework centered on multimodal data integration, advanced analytics, and real-time user interaction. As illustrated in Figure 3A, most systems follow a similar flow: data is acquired from diverse sources such as CGM, EHR, wearables, and multi-omics platforms; it is then processed through a suite of technological components, commonly cloud computing, IoT devices, edge AI, and mobile infrastructure, before being delivered to end users through mobile applications, dashboards, or clinical portals.

A central feature across these implementations is the predominant use of machine learning (ML) techniques over traditional physiological or compartmental models. The algorithm frequency cloud in Figure 3C emphasizes this shift, highlighting commonly used terms like “Machine Learning,” “LSTM,” “Bayesian,” “Prediction,” and “Neural Networks.” These algorithms are directly linked to the nature of the input data and the desired output. Figure 3B maps typical data-algorithm-output connections—demonstrating, for example, how CGM data is often processed by LSTM/RNN models to predict glucose trends, while wearable data is analyzed via Random Forest algorithms to estimate insulin needs or physical activity.

The review further reveals variability in system complexity and data update frequency, depending on the clinical application. As shown in Table 5, some digital twin systems deliver real-time predictions (e.g., every few minutes), while others update at daily, monthly, or episodic intervals. Applications range from tight glycemic control and lifestyle coaching to long-term risk stratification and metabolic modeling. Notably, studies such as those by Shamanna et al. (2020–2024) [14,15,18,19] and Joshi et al. (2023) [16] employ real-time integration of physiological signals, while others, like Surian et al. (2024) [22] and Zhang et al. (2024) [2], leverage broader clinical datasets for longitudinal monitoring.

Overall, while the core architecture and analytical models show convergence, the operationalization of digital twins in diabetes varies in data modalities, algorithmic complexity, and clinical scope, reflecting the flexibility and expanding frontiers of this emerging technology.

### 3.5. Performance and Clinical Outcomes

The included studies demonstrate both high technical performance and tangible clinical benefits, as summarized in Table 6. The predictive models included in this review reported robust performance, with key metrics such as the AUC often exceeding 0.85, and high Negative Predictive Values (NPV) for outcomes like chronic kidney disease, indicating a strong capacity to identify and predict relevant clinical events. Models developed for both type 1 (T1DM) and type 2 diabetes (T2DM) showed robust performance, with slightly higher accuracy reported in some T1DM-focused studies.

Regarding sample size, several models achieved high accuracy despite being trained on small populations, often with fewer than 50 participants. This suggests that the quality and physiological specificity of the data may compensate for limited cohort size, particularly when models are tailored to individual characteristics.

Beyond technical performance, a subset of studies evaluated direct clinical outcomes. Interventional trials reported significant reductions in HbA1c levels, ranging from −0.7% to −1.8%. In some cases, more transformative outcomes were observed, including T2DM remission, sustained weight loss, improved glycemic control (e.g., time in range and MARD), and positive changes in cardiovascular and hepatic biomarkers.

Validation approaches ranged from in silico simulations and retrospective analyses to more rigorous randomized controlled trials (RCTs), reflecting increasing methodological robustness in the assessment of these systems. This progression, combined with the integration of emerging technologies such as machine learning, IoT, and physiologically driven dynamic models, signals a clear advancement toward clinically meaningful digital twin applications in diabetes care.

### 3.6. System Integration and Personalization

**Figure 4:**
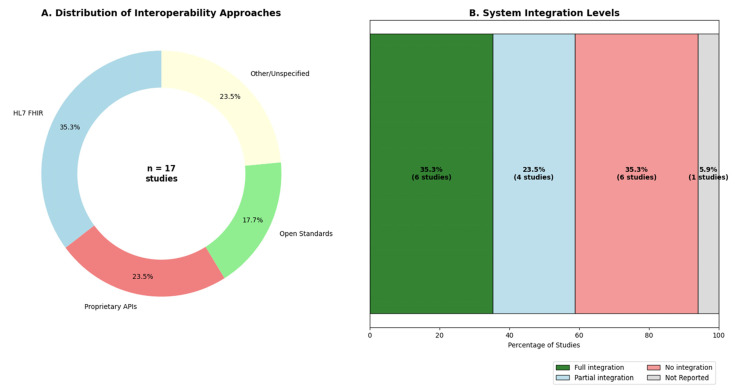
Interoperability landscape of diabetes digital twin systems. (**A**) The donut chart shows the distribution of interoperability approaches used in digital twin systems, with 35.3% adopting HL7 FHIR, 17.7% using other open standards, 23.5% relying on proprietary APIs, and 23.5% using other or unspecified methods. (**B**) The stacked bar chart presents the reported levels of system integration, revealing that 35.3% of studies achieved full integration, 23.5% had partial integration, 35.3% reported no integration, and 5.9% did not report integration status. Note: This figure was generated using Python.

Figure 4 illustrates the current landscape of interoperability approaches and integration levels in digital twin systems for diabetes. A common objective across studies is the seamless incorporation of digital twins into existing clinical workflows, particularly electronic health records (EHRs). However, implementation remains fragmented. As shown in Figure 4A, 35.3% of systems adopted HL7 FHIR standards, 17.7% used other open standards, and 23.5% relied on proprietary APIs. An additional 23.5% did not clearly specify their interoperability method. Integration levels also varied substantially, as depicted in Figure 4B. While 35.3% of systems reported full integration with clinical workflows, another 35.3% had no integration at all, and 23.5% reported only partial integration. These gaps underscore persistent barriers to widespread and standardized adoption. Despite these challenges, mobile applications remain the predominant interface for patient interaction. All systems described aim for personalization, applying methods that range from static user profiles to dynamic, adaptive algorithms that respond to an individual’s physiology, behavior, and, in some cases, multi-omics data, enabling a truly tailored approach to diabetes management.

### 3.7. Clinical Outcomes and Safety of Interventional Studies

A subset of the included articles were interventional studies that reported direct clinical outcomes. Table 7 provides a detailed overview of these key studies. The table outlines the study design, follow-up duration, participant characteristics, and primary efficacy outcomes, including reported effect sizes. The findings summarized in Table 7 show that digital twin interventions have been evaluated in various settings, from short-term retrospective analyses to one-year randomized controlled trials. The primary clinical outcomes reported are consistently positive, showing significant reductions in HbA1c, with mean changes ranging from −1.8% to −2.9% in year-long studies. Furthermore, the RCT led by Joshi, Shamanna et al. [16,19] reported high rates of T2D remission (72.7%) and hypertension remission (50%) in the digital twin group, outcomes not observed in the standard care arms. While efficacy outcomes are robustly reported, the table also highlights a critical gap: the inconsistent and often absent reporting of adverse events. Only one retrospective study explicitly mentioned non-serious, transient adverse events, while the larger RCTs did not report on them in the primary publications reviewed, a key limitation for assessing the overall safety profile of these interventions.

### 3.8. Interoperability and EHR Integration

To provide a more granular view of the implementation landscape, Table 8 details the interoperability methods, EHR integration levels, and reported barriers on a per-study basis. The analysis reveals a notable trend: conceptual frameworks often explicitly plan for open standards, such as the HL7 FHIR-aligned ontology proposed by Sarani Rad et al., or the large-scale database integration via knowledge graphs described by Zhang et al. In contrast, the published clinical intervention studies, while demonstrating successful device-level interoperability through IoT and proprietary platforms, do not typically report the use of formal healthcare standards or deep integration into existing EHR systems. This highlights a critical gap between conceptual architectures and real-world clinical implementation, where barriers shift from theoretical data standards to practical challenges like patient adherence and managing proprietary data streams.

## 4. Discussion

### 4.1. Principal Findings

Our comprehensive review reveals that digital twin research in diabetes represents a pivotal shift toward precision medicine, delivering notable clinical outcomes despite key barriers in implementation. The sharp increase in publications since 2022 (Figure 2A) indicates a growing global urgency to address the diabetes epidemic with more tailored and effective interventions. This momentum reflects not only academic curiosity but also a clinical imperative to move beyond generalized treatment approaches.

The geographic distribution of studies highlights a striking trend: India and the United States are leading innovation in digital twin technologies (Figure 2B). India, despite its high diabetes burden of over 77 million individuals, is also emerging as a hub for digital health innovation, driven by the convergence of public health need and technological capability.

Methodologically, the field is evolving from conceptual models (37.5%) to more mature designs, including randomized controlled trials (25%). This trend parallels broader developments in digital health and contrasts with findings from prior reviews in Type 1 diabetes, where validation on real-world data remains limited [4]. Our results suggest that digital twins for Type 2 diabetes are advancing faster in terms of clinical testing, likely due to the larger patient base and less variable insulin dynamics.

### 4.2. Technological Architecture: Convergence Toward Intelligent Personalization

We observed a convergence toward more sophisticated system architectures (Figure 3A), shifting from traditional mechanistic models to machine learning frameworks. LSTM networks and ensemble methods dominate (Figure 3C), well-suited for capturing the temporal complexity of glucose-insulin interactions. These models are increasingly fed by data from wearables, CGMs, and EHRs, enabling more accurate, real-time physiological modeling.

Crucially, a small but growing number of studies integrate multi-omics data, signaling a move toward deeply personalized care that accounts for individual genetic and metabolic profiles. Such efforts illustrate how digital twins are evolving into dynamic, adaptive tools capable of guiding precision interventions.

Yet, this technical progress is undermined by persistent interoperability challenges. Our per-study analysis in Table 8 reveals a significant gap: conceptual frameworks often propose open standards like HL7 FHIR, while the clinical intervention studies reviewed tend to rely on proprietary APIs and device-level connectivity. These fragmented standards limit interoperability, hinder scalability, and create barriers to integration across healthcare systems.

### 4.3. Clinical Impact: Transformative Outcomes Amid Implementation Complexities

Digital twins for diabetes are evolving beyond predictive tools to become potential drivers of tangible clinical benefits. The emerging evidence suggests that these systems can yield outcomes that may, in some contexts, surpass standard interventions ... Perhaps the most compelling finding from the reviewed literature is the reported potential for disease reversal; one notable randomized controlled trial reported T2D remission in 76.5% of its participants [17]. While this finding requires further validation in larger, multi-center studies, it suggests the potential to challenge the notion of Type 2 diabetes as a perpetually progressive condition.

Despite these promising outcomes, significant limitations persist that temper their current applicability. The majority of the reviewed studies were conducted at a single site and often with small sample sizes, which inherently limits the generalizability of the findings across different populations and healthcare systems. Furthermore, inconsistencies in how outcomes are reported make direct, cross-study comparisons difficult. Crucially, the evidence base is still maturing; of the 17 included studies, only three were randomized controlled trials, and the majority of the interventions reported follow-up periods of one year or less. This underscores the preliminary nature of the current evidence for clinical transformation.

A critical barrier remains in the final step: practical implementation. The integration of these sophisticated systems into established clinical workflows is markedly uneven. Furthermore, as detailed in Table 7, the reporting of safety data is a significant concern. The general lack of systematic reporting of adverse events in the reviewed interventional studies is a major gap that limits a comprehensive assessment of the safety profile of these digital twin interventions.

### 4.4. Comparative Analysis with Contemporary Literature: Positioning Our Findings

Our findings significantly extend and complement recent systematic analyses in digital health and precision medicine, while revealing unique characteristics of diabetes as a domain for digital twin implementation. The comprehensive healthcare digital twin review by Katsoulakis et al. (2024) [27] identified diabetes among eight major application domains but emphasized that “digital twins for health remain in early developmental stages” with substantial barriers to clinical translation. Our diabetes-specific analysis provides compelling counter-evidence, demonstrating that certain therapeutic domains may achieve mature implementation considerably earlier than others.

In contrast to the Type 1 diabetes systematic review by Cappon and Facchinetti (2024) [4], which identified significant limitations in real-world validation and predominantly simulation-based approaches, our analysis reveals that Type 2 diabetes digital twins have achieved robust clinical validation with substantial real-world evidence. This disparity likely reflects the greater physiological complexity and variability inherent in Type 1 diabetes management, where autoimmune pancreatic destruction creates more unpredictable glucose-insulin dynamics compared to the metabolic dysfunction characteristic of Type 2 diabetes.

The comprehensive umbrella review by Sun et al. (2024) [28] examining mobile phone interventions across chronic diseases, synthesizing 34 meta-analyses and 235 randomized controlled trials, reported that almost half (42%) of digital health intervention outcomes were non-significant, with only one outcome achieving “convincing” evidence (mobile apps for HbA1c reduction in Type 2 diabetes, d = −0.44). This contrasts sharply with our findings of consistent, clinically meaningful improvements across multiple diabetes digital twin studies, with HbA1c reductions ranging from −0.7% to −1.9% and sustained clinical benefits. This suggests that digital twins may represent a qualitatively different intervention paradigm compared to traditional digital health tools, potentially due to their capacity for dynamic, personalized modeling rather than static guideline implementation.

The economic evaluation framework proposed by Zhang et al. (2024) [2] for digital health implementations emphasized the critical importance of demonstrating both clinical effectiveness and economic sustainability. Our review identifies significant gaps in economic evaluation among diabetes digital twins, with only 23.5% of studies reporting cost-effectiveness data despite demonstrating substantial clinical benefits that theoretically should translate to healthcare cost reductions through complication prevention.

### 4.5. Critical Limitations and Methodological Considerations

Despite rigorous adherence to PRISMA-ScR guidelines and comprehensive search strategies across multiple databases, our review has inherent limitations that must be acknowledged. The predominant geographic concentration in India, while providing methodological consistency, may limit generalizability to populations with different genetic backgrounds, healthcare systems, and socioeconomic contexts. The exclusion of non-English publications potentially underrepresents innovations from other regions, particularly given the global nature of diabetes as a health challenge.

Our study adheres to a scoping review methodology; therefore, a quantitative meta-analysis was not an objective. The considerable heterogeneity in study designs (from RCTs to in silico simulations), populations, and specific outcomes reported, as detailed in our summary tables, would in any case preclude such a synthesis. Consequently, we employed a narrative synthesis, which, while appropriate, may be subject to interpretive bias. Additionally, the rapid evolution of digital twin technology means that some analyzed studies may already represent outdated approaches, while emerging innovations remain unpublished or in development phases.

Publication bias toward positive results is a persistent concern in digital health research, potentially inflating perceived effectiveness. The predominance of studies from single research groups, particularly the Shamanna consortium, while methodologically advantageous for consistency, may limit the diversity of approaches and validation across different populations and healthcare contexts.

Given the significant contribution of a single research consortium to the evidence base on clinical outcomes, a sensitivity analysis is warranted. A narrative exclusion of the studies conducted by Shamanna et al. [13,14,15,16,17,18,19] would fundamentally alter the conclusions of this review regarding Type 2 Diabetes. Without these studies, the evidence for tangible clinical outcomes, such as T2DM remission and substantial HbA1c reductions, would be nearly absent. The focus of the remaining literature would shift dramatically towards conceptual frameworks, technical validation of in silico models, and applications predominantly for Type 1 Diabetes. This sensitivity analysis reveals that the current evidence for high-impact clinical applications of digital twins in T2D is heavily reliant on the findings of this single group. While this highlights their pioneering work, it also underscores a critical limitation of the current landscape: a lack of independent replication. Therefore, a key direction for future research must be the validation of these transformative outcomes by independent research groups across different populations and technological platforms to ensure the generalizability of these findings

### 4.6. Implications for Clinical Practice: Bridging Innovation and Implementation

The implementation of digital twins in clinical practice faces critical barriers. Fragmented interoperability, as shown in our analysis (Figure 4A), where only 35.3% of systems adopt open standards such as HL7 FHIR (Figure 4A), hampers integration with existing electronic health records and clinical workflows [7,29]. This limitation promotes isolated solutions that increase administrative burden and hinder proactive continuous monitoring, which is essential in diabetes management. Although tangible clinical benefits have been documented, such as HbA1c reductions of up to 1.9%, the absence of cost-effectiveness evaluations overlooks economic realities [30]. The required infrastructure, including IoT, cloud services, sensors, and maintenance of machine learning algorithms, entails substantial investments that remain unquantified. Without sustainable financial models, scalability in resource-limited health systems is unfeasible [31].

It is imperative to develop comprehensive clinical decision support systems that integrate digital twin predictions into current workflows [32]. This requires not only technological tools but also a paradigm shift in decision-making, moving from reactive, episodic care to continuous monitoring and proactive intervention [33]. While digital twins could theoretically reduce healthcare costs by preventing complications and optimizing disease management, initial investments and ongoing operational expenses demand rigorous economic evaluation to ensure alignment with healthcare financing models [34].

Clinical adoption also depends on overcoming challenges related to patient engagement and regulatory frameworks. Continuous data collection, such as daily glucose measurements, requires high adherence, yet most studies fail to assess user experience or trust in algorithmic recommendations [35]. This omission is critical, as distrust or technological fatigue may negate the utility of otherwise robust systems. Additionally, there is a regulatory gap, with 70.6% of studies conducted in non-experimental research contexts (Figure 2C), and no clear pathways for the clinical approval of adaptive algorithms that learn in real time [36].

### 4.7. Future Research Directions: Toward Mature Digital Twin Ecosystems

While this review confirms the burgeoning potential of digital twins in diabetes, it also illuminates a clear path for future research required to transition from promising, often isolated, demonstrations to mature, scalable, and validated clinical ecosystems. The current evidence base, though encouraging, is a foundation upon which a more robust scientific structure must be built. Future research should be strategically directed toward addressing several key gaps.

First and foremost, there is a critical need for longitudinal, multi-center randomized controlled trials. While the studies included report impressive short-term outcomes, such as T2DM remission and significant HbA1c reductions, the long-term sustainability of these benefits remains unproven. Future investigations must extend beyond 12-month follow-ups to assess the durability of remission, the long-term impact on micro- and macrovascular complications, and mortality rates. Conducting these trials across diverse geographical and healthcare settings is paramount to establishing the external validity and generalizability of digital twin interventions, moving beyond the current concentration in specific regions like India.

Secondly, comparative effectiveness research is essential. To date, most studies compare digital twin interventions against standard care. A critical next step involves head-to-head comparisons against other high-intensity interventions, including other digital health applications (e.g., simpler coaching apps), intensive pharmacological regimens, or bariatric surgery. Such studies would clarify the unique value proposition of the digital twin approach and help define its precise place in the clinical armamentarium. This should be coupled with rigorous health economic analyses, a domain largely absent in the current literature. Robust cost-effectiveness and budget impact models are indispensable for justifying reimbursement and securing investment from healthcare systems.

Moreover, the technological frontier must continue to be advanced through research into hybrid modeling and multi-omics integration. While machine learning models currently dominate, hybrid approaches that combine the predictive power of ML with the interpretability of mechanistic physiological models could offer the best of both worlds: accuracy and explainability. Furthermore, as conceptualized by Zhang et al. (2024) [2], the practical integration of multi-omics data (genomics, proteomics, metabolomics) into dynamic digital twins represents the next evolutionary step. Research must focus on developing computationally efficient methods to translate this high-dimensional data into clinically actionable insights for true N-of-1 precision medicine.

A greater emphasis must be placed on human-centered design and implementation science. The most sophisticated algorithm is ineffective if the patient-facing interface is burdensome or clinician workflow integration is clumsy. Future studies should incorporate formal usability testing, explore factors driving long-term patient engagement and adherence, and assess the cognitive load on both patients and healthcare providers. Understanding these human factors is not a secondary concern but a core component of translating technological innovation into real-world clinical impact.

### 4.8. Regulatory and Ethical Imperatives: Ensuring Responsible Innovation

In the ethical-regulatory domain, urgent risks emerge. The findings of this study underscore the necessity of validating diagnostic criteria and predictive models within the target population before clinical application, a requirement equally relevant to digital twins. Just as we found that cut-off points developed in other regions may not accurately reflect local body composition, algorithms trained in geographically limited contexts risk reproducing structural biases [37].

The overrepresentation of Indian populations (58.8% of studies, Figure 2B) and specific age groups (Table 4) generates algorithmic biases that may exacerbate health disparities by underrepresenting pediatric, geriatric, or minority ethnic populations [38]. The opacity of “black box” models, used in 76% of systems (Figure 3C), undermines clinical transparency: only two studies incorporate explainable AI (XAI) techniques, hindering the validation of critical recommendations such as insulin adjustments and limiting robust informed consent [38]. There is a lack of defined legal liability in the event of errors; for example, hypoglycemia caused by algorithmic failures remains unresolved and discourages clinical adoption [39].

Data governance must also evolve toward dynamic models. The continuous flow of sensitive information, arising from CGM, EHR, or genomics, conflicts with the static consent frameworks used in 100% of studies. It is urgent to implement granular and revocable consent, enabling patients to control secondary uses of their data in real time. Without advances in standardized interoperability, such as HL7 FHIR as a requirement, rigorous economic evaluation, bias mitigation through multiracial validation, and adaptive ethical frameworks, the transformative potential of digital twins will remain confined to fragmented or inequitable settings [40].

The ethical management of patients’ bodily and functional information is non-negotiable. This highly sensitive resource requires governance systems that ensure confidentiality, patient control, and protection against unauthorized use. The adoption of standards such as “dynamic consent” can balance the scientific use of data with respect for individual autonomy, supporting a technological implementation that is both innovative and socially acceptable [40].

## 5. Conclusions

This scoping review successfully mapped the rapidly advancing landscape of digital twins in diabetes, revealing a field that has matured from conceptual frameworks to clinically impactful applications. In fulfillment of our objective, we found that current implementations predominantly leverage IoT-driven architectures and machine learning models to integrate multimodal data for highly personalized predictions. These systems have demonstrated remarkable clinical results in some studies, achieving significant improvements in glycemic control and, in some cases, T2DM remission, thus presenting findings that could challenge traditional paradigms of chronic disease management. However, this review also reveals that this technological progress is contrasted by critical gaps: the evidence for high-impact T2DM outcomes relies heavily on a single research consortium, systematic safety data is largely absent, economic evaluations are almost entirely missing, and deep integration into clinical workflows remains the exception rather than the rule. Therefore, while digital twins have proven their potential to revolutionize diabetes care, translating this potential into standard practice hinges on independent validation of clinical outcomes, the standardization of interoperability frameworks, and rigorous assessment of long-term safety and cost-effectiveness in diverse populations.

## Figures and Tables

**Table 1:** Eligibility criteria.

Inclusion Criteria	Exclusion Criteria
**Study Types:**	**Study Types:**
• Empirical studies (observational, experimental, modeling)	• Studies without empirical data, systematic or scoping reviews or metanalysis
	• Conceptual and theoretical models that lack a detailed architectural framework or a clear pathway for future clinical validation
• Original research published in peer-reviewed journals	• Opinion articles, editorials, commentaries
• Full-text articles available	• Conference abstracts, preprints, theses
• English	• Brief communications without original data
**Population:**	**Population:**
• Human patients with diabetes mellitus (any type)	• Non-human studies (animal models, in vitro)
• Type 1, Type 2, gestational, or other diabetes variants	• Studies not focused on diabetic populations
• Any age group (pediatric, adult, elderly)	• Healthy populations without diabetes
• Any geographical location	• Mixed populations where diabetes data cannot be extracted
**Concept/Technology:**	**Concept/Technology:**
• Digital twins implementation in diabetes management	• Traditional monitoring systems only
• Virtual patient models with real-time data integration	• Studies without digital twin components
• Predictive modeling for diabetes complications	• Simple mobile apps without modeling capabilities
• Personalized treatment optimization systems	• Basic data collection tools without analytics
• Integration of wearable devices, CGM, IoT sensors	• Single-parameter monitoring systems
• Machine learning/AI applications in diabetes care	• Static risk calculators
**Outcomes/Context:**	**Outcomes/Context:**
• Clinical effectiveness metrics reported	• Unclear or insufficient outcome data
• Performance measures (sensitivity, specificity, accuracy)	• Studies with incomplete results
• Integration with traditional healthcare systems	• Purely technical/engineering focus without clinical application
• Real-world implementation evidence	• Simulation studies without real patient data
• Patient-reported outcomes and acceptability	• Studies focusing solely on system architecture
• Cost-effectiveness or feasibility data	• No validation against clinical standards

**Table 2:** Data extraction variables.

Variable Category	Specific Variables Extracted
**Bibliographic and Methodological Information**	
	• Author names and publication year
	• Study design and duration
	• Geographical location and setting
	• Sample size and power calculations (when available)
	• Demographic characteristics of study populations
**Population Characteristics**	
	• Diabetes type (type 1, type 2, gestational, monogenic, other variants)
	• Age distribution and sex composition
	• Diabetes duration and disease severity
	• Comorbidity profiles and complications history
	• Previous pharmacological treatments and management approaches
**Digital Twin Implementation Variables**	
	• Data sources: continuous glucose monitors, flash glucose monitoring systems
	• Wearable activity trackers, smart insulin pens, mobile health applications
	• Electronic health record integration
	• System architecture and technological platforms
	• Cloud computing infrastructure, edge computing capabilities
	• Data processing frameworks
	• Analytical algorithms: machine learning (random forests, SVM, neural networks)
	• Statistical models (time series analysis, regression methods)
	• Deep learning architectures and ensemble modeling techniques
**Performance and Validation Metrics**	
	• Predictive accuracy: sensitivity, specificity, PPV, NPV
	• Area under receiver operating characteristic curve (AUC-ROC)
	• Calibration metrics
	• Correlations with traditional methods (HbA1c, SMBG, clinical assessments)
	• Temporal advantages: lead time measurements, early warning capabilities
	• Glycemic control: time in range, time above/below range
	• Glycemic variability indices, mean absolute relative difference
**Integration and Implementation Variables**	
	• Validation methods: cross-validation, external validation, real-world evidence
	• EHR compatibility, clinical decision support integration
	• Healthcare provider workflow incorporation
	• Impact on clinical decisions and treatment adherence
	• Patient-reported outcomes and quality of life measures
	• Healthcare utilization patterns
**Contextual and Feasibility Variables**	
	• Spatial and temporal data granularity specifications
	• Ethical considerations and privacy protection measures
	• Regulatory compliance and approval status
	• Technological barriers and implementation challenges
	• User acceptability (patients and healthcare providers)
	• Training requirements for clinical staff
	• Cost-effectiveness analyses and budget impact assessments

T2DM: type 2 diabetes mellitus; GDM: gestational diabetes mellitus; CGM: continuous glucose monitoring; FGM: flash glucose monitoring; mHealth: mobile health; EHR: electronic health record; ML: machine learning; SVM: support vector machines; NN: neural networks; DL: deep learning; PPV: positive predictive value; NPV: negative predictive value; AUC-ROC: area under receiver operating characteristic curve; HbA1c: hemoglobin A1c; SMBG: self-monitoring blood glucose; TIR: time in range; TAR: time above range; TBR: time below range; GV: glycemic variability; MARD: mean absolute relative difference; RWE: real-world evidence; CDS: clinical decision support; PRO: patient-reported outcomes; QoL: quality of life.

**Table 3:** Bibliographic information.

Authors	Year	Title	Journal	Country	Study Type
Shamanna et al. [13]	2020	Reducing HbA1c in Type 2 Diabetes Using Digital Twin Technology-Enabled Precision Nutrition: A Retrospective Analysis	Diabetes Therapy	India	Retrospective
Shamanna et al. [14]	2021	Retrospective study of glycemic variability, BMI, and blood pressure in diabetes patients in the Digital Twin Precision Treatment Program	Scientific Reports	India	Retrospective cohort
Shamanna et al. [15]	2021	Type 2 diabetes reversal with digital twin technology-enabled precision nutrition and staging of reversal: a retrospective cohort study	Clinical Diabetes and Endocrinology	India	Retrospective cohort
Joshi, Shamanna et al. [16]	2023	Digital Twin-Enabled Personalized Nutrition Improves Metabolic Dysfunction-Associated Fatty Liver Disease in Type 2 Diabetes: Results of a 1-Year Randomized Controlled Study	Endocrine Practice	India	RCT
Shamanna et al. [17]	2024	Personalized nutrition in type 2 diabetes remission: application of digital twin technology for predictive glycemic control	Frontiers in Endocrinology	India	RCT
Shamanna et al. [18]	2024	One-year outcomes of a digital twin intervention for type 2 diabetes: a retrospective real-world study	Scientific Reports	India	Retrospective
Shamanna et al. [19]	2024	Digital twin in managing hypertension among people with type 2 diabetes 1-year randomized controlled trial	JACC: Advances	India	RCT
Colmegna et al. [20]	2020	Mapping data to virtual patients in type 1 diabetes	Control Engineering Practice	Spain/USA	In silico simulation
Hughes et al. [21]	2021	Replay Simulations with Personalized Metabolic Model for Treatment Design and Evaluation in Type 1 Diabetes	Journal of Diabetes Science and Technology	USA	In silico validation
Cappon et al. [4]	2023	ReplayBG: A Digital Twin-Based Methodology to Identify a Personalized Model From Type 1 Diabetes Data and Simulate Glucose Concentrations to Assess Alternative Therapies	IEEE Transactions on Biomedical Engineering	Italy	Technical validation
Sarani Rad et al. [1]	2024	Personalized Diabetes Management with Digital Twins: A Patient-Centric Knowledge Graph Approach	Journal of Personalized Medicine	USA	Conceptual framework
Surian et al. [22]	2024	A digital twin model incorporating generalized metabolic fluxes to identify and predict chronic kidney disease in type 2 diabetes mellitus	NPJ Digital Medicine	Australia/Singapore	Model development
Zhang et al. [2]	2024	A framework towards digital twins for type 2 diabetes	Frontiers in Digital Health	China/USA	Conceptual framework
Visentin et al. [23]	2016	One-Day Bayesian Cloning of Type 1 Diabetes Subjects: Towards a Single-Day UVA/Padova Type 1 Diabetes Simulator	IEEE Transactions on Biomedical Engineering	Italy	Model development
Thamotharan et al. [24]	2023	Human digital twin for personalized elderly type 2 diabetes management	Journal of Clinical Medicine	India	Conceptual
Young et al. [25]	2024	Design and In Silico Evaluation of an Exercise Decision Support System Using Digital Twin Models	Journal of Diabetes Science and Technology	USA	In silico evaluation
Deichmann et al. [26]	2023	New model of glucose-insulin regulation characterizes effects of physical activity and facilitates personalized treatment evaluation in children and adults with type 1 diabetes	PLOS Computational Biology	Switzerland	Model development

**Table 4:** Population characteristics.

Authors	Diabetes Type	Sample Size	Age (Years)	Female (%)	Diabetes Duration	Comorbidities	Baseline HbA1c (%)	Key Inclusion/Exclusion Criteria
Shamanna et al. [13]	T2DM	64	52.4 ± 10.0	29.7%	8.4 ± 6.5 years	Adequate hepatic/renal function required	8.8 ± 2.2	Incl: T2D, adequate hepatic/renal function.
Shamanna et al. [14]	T2DM	64	52.44 ± 9.9	29.69%	8.43 ± 6.5 years	No recent cardiovascular events	8.8 ± 2.2	Incl: T2D, adequate hepatic/renal function.
Shamanna et al. [15]	T2DM	463–475	48.5 ± 10.6	27.4%	9.1 ± 7.5 years	Adequate hepatic/renal function	9.0 ± 1.9	Incl: T2D, adequate hepatic/renal function.
Joshi, Shamanna et al. [16]	T2DM	319	18–70	Not reported	≥8 years	Normal hepatic/renal function	DT: 9.0 ± 1.9 SC: 8.5 ± 1.9	Incl: Age 18–70, T2D < 8 years, normal hepatic/renal function.
Shamanna et al. [17]	T2DM	319	18–70	Not reported	≤8 years	HTN, MAFLD, CVD, neuropathy	DT: 9.0 ± 1.9 SC: 8.5 ± 1.9	Incl: Age 18–70, T2D < 8 years.
Shamanna et al. [18]	T2DM	1853	50.9 ± 9.9	20.5%	6.7 ± 6.2 years	Obesity (36.9%), HTN (38.2%), NAFLD (83.1%)	8.1 ± 1.7	Incl: Age 18–80, T2D diagnosis.
Shamanna et al. [19]	T2DM	319	DT: 43.6 ± 8.6, SC: 51.8 ± 10.5	DT: 16.3%, SC: 53.5%	3.8 ± 2.6 years	Hypertension	DT: 9.0 ± 1.9 SC: 8.4 ± 1.9	Incl: Age 18–70, T2D ≤ 8 years, normal hepatic/renal function.
Colmegna et al. [20]	T1DM	15	41 ± 11	73%	25 ± 10 years	None reported	7.41 ± 0.97	Participants from a previous clinical trial (NCT02558491).
Hughes et al. [21]	T1DM	100 (virtual)	Adult virtual subjects	N/A	N/A	N/A (simulation)	N/A	N/A (in silico study)
Cappon et al. [4]	T1DM	100 (virtual) + 1 real	Adult virtual subjects	N/A	N/A	N/A (simulation)	N/A	N/A (in silico study)
Sarani Rad et al. [1]	Not specified	N/A (model)	N/A	N/A	N/A	N/A (conceptual framework)	N/A	N/A (conceptual framework)
Surian et al. [22]	T2DM	7072	EVAS: 54 ± 11.1, NHANES: 59 ± 11.9, SDR: 61.2 ± 11.0, CDMD: 57 ± 12.4	~48–50%	Not reported	Hypertension, CKD	EVAS: 8.6 ± 1.8 NHANES: 7.6 ± 1.9 SDR: 7.4 ± 1.6 CDMD: 8.0 ± 1.8	T2DM patients from four existing multi-ethnic cohorts, aged 20–80.
Zhang et al. [2]	T2DM	1131	49.53 ± 11.29	Not reported	Not applicable	CKD progression focus	5.51 ± 0.43	Participants from Arivale dataset with multi-omic data.
Visentin et al. [23]	T1DM	47	42.0 ± 10.1	Not reported	Not reported	Not reported	Not reported	T1DM subjects from the AP@home FP7-EU project clinical trial.
Thamotharan et al. [24]	T2DM	15	36–80	53.3%	Not reported	HTN, dyslipidemia, CKD, heart disease	Not reported	Elderly T2D patients with comorbidities recruited at JDRC, India.
Young et al. [25]	T1DM	247	Not reported	Not reported	Not reported	Not reported	N/A	Digital twins matched to T1Dexi study participants based on insulin sensitivity and weight.
Deichmann et al. [26]	T1DM	5	ago-14	Not reported	Not reported	Not reported	Not reported	Children with T1D from the ‘DiaActive’ study.

Abbreviatures: HTN = hypertension; CKD = chronic kidney disease; NAFLD = non-alcoholic fatty liver disease; MAFLD = metabolic dysfunction-associated fatty liver disease; CVD = cardiovascular disease; DT = digital twin; SC = standard care.

**Table 5:** Digital twin implementation.

Authors	Technological Components	Algorithms/Models	Data Sources	Captured Parameters	Update Frequency
Shamanna et al. [13]	IoT, Cloud, Mobile app, Wearables (Fitbit), Bluetooth devices	Machine learning (gradient boosted trees, deep learning, LSTM)	CGM (Abbott Libre Pro), Fitbit, BP monitor, smart scale, food app	Glucose (96/day), activity, sleep, weight, BP, ketones, nutrition	Daily
Shamanna et al. [14]	Digital platform, TPT system, IoT sensors, Mobile app	Machine learning algorithms, precision nutrition algorithm	CGM, wearables, clinical data, food logging	Glucose, activity, nutrition, clinical biomarkers	Daily
Shamanna et al. [15]	Digital twin platform, Mobile app, Cloud-based	ML algorithms, clustering models	CGM, clinical assessments, nutrition data	Glucose, HbA1c, insulin resistance markers	90-day intervals
Joshi, Shamanna et al. [16]	Digital twin platform, Mobile devices, Wearables	ML, precision nutrition algorithms	CGM, wearables, mHealth devices	>100 physiological signals, glucose, liver markers	Real-time to monthly
Shamanna et al. [17]	DT platform, Mobile app, Wearables	ML algorithms, predictive models	CGM, wearables, clinical data	Glucose, physiological markers, lifestyle	Real-time
Shamanna et al. [18]	Digital platform, Mobile app, IoT devices	ML algorithms, data analytics	CGM, wearables, clinical assessments	Glucose, weight, BP, activity, nutrition	Daily to monthly
Shamanna et al. [19]	Twin Health platform, Mobile app, Sensors	ML, AI algorithms	CGM, wearables, clinical data, BP monitoring	Glucose, BP, weight, activity	Real-time
Colmegna et al. [20]	UVA/Padova simulator, HPC systems, Matlab toolbox	Mathematical model, Fourier series, optimization	CGM, insulin pumps, meal data	Glucose, insulin, carbohydrates, 8 physiological parameters	5 min sampling, daily parameter updates
Hughes et al. [21]	UVa/Padova simulator, Computational platform	SOGMM, LTI model, regularized deconvolution	In silico data, CGM, insulin/meal records	Glucose, insulin sensitivity, meal absorption parameters	Per day (SI, f), per meal (absorption)
Cappon et al. [4]	UVa/Padova simulator, Digital twin framework	Personalized metabolic models, deconvolution algorithms	CGM, insulin pumps, carbohydrate intake	Glucose, insulin, carbohydrates, physiological parameters	Daily parameter identification
Sarani Rad et al. [1]	Knowledge graph platform, Digital twin framework	Machine learning, knowledge graphs	PHKGs, clinical data, IoT sensors	Patient health knowledge, glucose patterns	Variable/real-time
Surian et al. [22]	Cloud-based ML pipeline, HealthVector Diabetes^®^	Logistic regression, GMF-based models	EHR, clinical labs, multi-ethnic cohorts	33 clinical variables, metabolic fluxes	Episodic (yearly aggregation)
Zhang et al. [2]	Multilevel system, Cloud platform	Neural networks, deep learning, multi-omics integration	Multi-omics data, clinical records, longitudinal data	>200 integrated variables, biomarkers	Variable
Visentin et al. [23]	UVA/Padova simulator, Bayesian framework	Bayesian identification, compartmental model	Clinical trial data, glucose/insulin measurements	Glucose, insulin, model parameters	Single-day identification
Thamotharan et al. [24]	Mobile platform, IoT sensors, Cloud	Digital twin algorithms, personalized models	Clinical data, sensor data, patient records	Glucose, clinical parameters, lifestyle factors	Variable
Young et al. [25]	Digital twin simulator, Exercise DSS, OHSU metabolic simulator	Digital twin models, exercise algorithms	Heart rate, insulin, meal data, T1Dexi dataset	Glucose, exercise parameters, insulin	30 min exercise sessions
Deichmann et al. [26]	Computational platform	Mathematical glucose-insulin regulation model	CGM, accelerometer, logbook data	Glucose, insulin, physical activity, carbohydrates	Multi-day continuous

Abbreviatures: CGM = continuous glucose monitoring; IoT = Internet of Things; ML = machine learning; LSTM = Long Short-Term Memory; BP = blood pressure; HPC = High-Performance Computing; EHR = electronic health record; GMF = Generalized Metabolic Fluxes; SOGMM = Subcutaneous Oral Glucose Minimal Model; LTI = Linear Time-Invariant; DSS = Decision Support System.

**Table 6:** Performance metrics.

Authors	Clinical Metrics	Validation Type	System Integration	User Interface	Interoperability	Personalization
Shamanna et al. [13]	HbA1c (−1.9%), Weight (−4.8 kg), TIR maintained 87.1%, HOMA-IR (−56.9%)	Retrospective	TPN system integrates CGM (Abbott Libre Pro), Fitbit Charge 2, Bluetooth BP monitor (TaiDoc TD-3140), smart scale (PowerMax BCA-130), secure cellular network transmission	TPN mobile app for food logging (>2000 USDA foods), biometric feedback, interaction with nutritional coaches	Compatible with commercial health devices, integration with USDA FoodData Central databases	ML algorithms analyze CGM and food intake for personalized PPGR predictions, individualized dietary recommendations without caloric limits
Shamanna et al. [14]	HbA1c reduction, BMI decrease, BP reduction, TIR > 70%, CV 17.34 ± 4.35%	Retrospective	TPT platform integrates body sensors (Fitbit Charge 2), Bluetooth BP monitor (TAIDOC TD-3140), smart scale (Powermax BCA-130), CGM (Abbott Libre Pro), secure cellular transmission	Twin mobile app for food logging (>2000 foods), biometric data visualization, health coach assistance	Interoperability with multiple devices from different brands via Bluetooth and cellular networks, integration with external nutritional databases	Dynamic personalized digital twin with ML, individual PPGR predictions, daily nutrition, exercise and sleep recommendations adapted to specific metabolism
Shamanna et al. [15]	Diabetes reversal staging progression	Retrospective	TPN system integrates CGM, sensor watches, BP monitors, smart scales, detailed food intake logging via mobile app, transmission to web platform	Mobile app with database of >50,000 foods with complete nutritional values, nutritional guidance by coaches via app and telephone	Integration with multiple data sources (CGM, sensors, blood analysis) through IoT technologies	ML analyzes macronutrients, micronutrients, biota nutrients for personalized PPGR predictions, dynamic representation of patient-specific metabolism
Joshi, Shamanna et al. [16]	HbA1c improvement, MAFLD markers improvement, MRI-PDFF reduction	RCT	DT system integrates CGM (Abbott FreeStyle Libre Pro), smartwatch (Fitbit Charge 2), smart scale (Powermax BCA-130), BP monitor (TAIDOC TD-3140), secure cellular transmission	Mobile app with color-coded foods (red/yellow/green), real-time recommendations, communication with health coaches	Integration with IoT devices from different manufacturers, processing via ML (gradient-boosted decision trees, deep learning, LSTM)	Individualized PPGR predictions, personalized food recommendations based on specific glycemic response, adaptive treatment phases
Shamanna et al. [17]	T2D remission (76.5%), improved glycemic control	RCT	DT platform integrates CGM, IoT devices, activity/sleep sensors, BP monitors, scale, secure cellular transmission	Twin app (WBDT) with technological nudge system, remote health coach assistance	IoT integration with >200 feature analysis via ML, compatibility with multiple commercial devices	AI-driven personalization with PPGR predictions, adaptive color coding, recommendations specific to individual moment and condition
Shamanna et al. [18]	HbA1c (−1.8%), Weight (−4.8 kg), TIR (69.7% to 86.9%), 74% medication reduction	Retrospective	DT system integrates CGM (Abbott FreeStyle Libre Pro), smartwatch (Fitbit Charge 2), smart scale (Powermax BCA-130), continuous data transmission via cellular network	Twin app with Daily Action Score aggregating health modules (sleep, breathing, activity, nutrition), simplified mobile interface	Automatic synchronization with multiple health devices, cross-validation between biometric sources	Personalized ML for >200 variables, dynamic recommendations adapted to individual metabolic profile, non-intrusive nudge system
Shamanna et al. [19]	BP reduction, hypertension management improvement	RCT	DT platform integrates multiple health devices (CGM, smartwatch, scale, BP monitor) via IoT sensors, secure cellular transmission	App with real-time technological nudges, color-coded foods, remote coach assistance	IoT connectivity with data exchange between devices and systems, integrated ML analysis	Personalized PPGR predictions, individual-specific color-coded food recommendations, adaptive phase-based approach
Colmegna et al. [20]	MARD < 10%, improved glucose prediction accuracy	In silico simulation	Integration of field-collected data (CGM, insulin pump, meal records) with time-invariant UVA/Padova model plus variability component	Conceptual/simulation system without described end-user interface	Interoperability with UVA/Padova simulator, compatibility with standard commercial device data	Personalization through identification of most sensitive UVA/Padova model parameters and subject-specific variability component
Hughes et al. [21]	Model validation metrics, glucose prediction accuracy	In silico validation	Personalized simulator integrated with UVa/Padova T1DM data, individualized physiological parameter identification	No specific user interface described, research simulation system	Integration with UVa/Padova simulator, acceptance of real clinical data for personalized analysis	Strong personalization through physiological parameter adjustment (insulin sensitivity, carbohydrate absorption) specific per subject and day
Cappon et al. [4]	ReplayBG validation, accurate therapy simulation	Technical validation	ReplayBG (University of Padova, Padova, Italy) open-source software in MATLAB (Mathworks Inc., Natick, MA, USA), two-step methodology for personalized model identification and simulation	No graphical interface described, available as MATLAB code on GitHub	Open-source software, compatible with standard CGM/insulin/carbohydrate data, interoperability via common data formats	Advanced personalization through Bayesian identification of individual non-linear glucose-insulin dynamics models specific per patient
Sarani Rad et al. [1]	Accurate glucose prediction and control	Conceptual validation	Framework integrates EHRs, wearable devices, mobile health apps, patient-generated data using GLAV framework	Healthcare Data Digital Twin Explorer with keyword search and dropdown tree navigation	Ontology aligned with HL7 FHIR standards for interoperability, integration with multiple heterogeneous data sources	Adaptive PHKGs (Personal Health Knowledge Graphs) that expand with new patient data, personalized meal recommendations
Surian et al. [22]	CKD prediction, NPV: 84–85%, PPV: 65–66%	Internal/External validation	GMF model integrates complete and incomplete clinical data from multiple datasets (EVAS, NHANES, SDR, CDMD)	Standard implementation in commercial tool “HealthVector Diabetes^®^”, graphical visualization of GMF profiles	Interoperability with multiple data sources, automatic handling of missing parameters without imputation	Personalized digital twin based on individual biological data, prediction of patient-specific disease trajectories
Zhang et al. [2]	Framework validation for T2D progression	Conceptual framework	Framework integrates ML, knowledge graphs (SPOKE with 41 databases), mechanistic models for multi-omic analysis	Future dashboard and UI suggested with natural language interfaces and LLMs	Interoperability through SPOKE graph consolidating 41 biomedical databases	Personalized design with different abstraction levels adapted to specific applications, individualized monitoring and intervention
Visentin et al. [23]	Bayesian parameter identification accuracy	Model validation	UVA/Padova simulator with mathematical model, in silico population, interface for scenario configuration	Interface mentioned for simulation scenario setup, running tools, displaying/saving results	No specific interoperability with external systems mentioned	Personalization through individual Bayesian cloning, incorporation of diurnal and inter-individual variability in glucose absorption and insulin sensitivity
Thamotharan et al. [24]	Personalized glucose management	Case studies	HDT framework integrates IoT/IoMT devices (wearable sensors, CGM), edge computing (Raspberry Pi), cloud services	Flask/HTML web interface with data visualization, mobile app for real-time vital signs monitoring	Integration of multiple sensor types (BLE, NFC, WiFi, RF), automatic device discovery by UUID	Adaptive patient model with ML (LSTM, predictive control), personalization of insulin infusion, activity/food recommendations
Young et al. [25]	Exercise DSS safety and efficacy validation	In silico evaluation	exDSS system integrates CGM, insulin pumps, meal logs, heart rate monitors, digital twins from OHSU metabolic simulator	No detailed user interface described, pre-exercise recommendation system	Functional interoperability between diverse medical devices (CGM, pumps, heart monitors)	Personalized digital twins calibrated by insulin sensitivity and weight, context-specific recommendations per individual physiological condition
Deichmann et al. [26]	Model fit to clinical data, glucose-insulin dynamics	Model validation	Modular model integrates CGM data, accelerometers, meal/insulin logs, compatible with existing pharmacokinetic modules	Open-source Python code available, no end-user interface described	Compatible with everyday device data (CGM, activity trackers), modular structure for integration	Robust personalization through least squares regression for individual parameters (insulin sensitivity, meal absorption, basal glucose)

Abbreviatures: MARD = mean absolute relative difference; TIR = time in range.

**Table 7:** Summary of interventional study designs, outcomes, and safety data.

Authors	Study Design & Follow-Up	Baseline & Key Inclusion Criteria	Key Clinical Outcomes (Effect Size)	Adverse Events Reported
Joshi, Shamanna et al. [16,19]	RCT 1-year follow-up	N = 319. T2D < 8 years, age 18–70, normal hepatic/renal function. Baseline HbA1c: 9.0% (DT) vs. 8.5% (SC).	• HbA1c change: −2.9% (DT) vs. −0.3% (SC), *p* < 0.001 • T2D Remission: 72.7% in DT group • HTN Remission: 50% in DT group vs. 0% in SC, *p* < 0.0001	Not explicitly reported
Shamanna et al. [18]	Retrospective 1-year follow-up	N = 1853. T2D, age 18–80. Baseline HbA1c: 8.1%.	• HbA1c change: −1.8% (SD 1.7), *p* < 0.001 • Weight change: −4.8 kg (SD 6.0), *p* < 0.001 • Medication reduction: 74%	Not reported
Shamanna et al. [13]	Retrospective 90-day follow-up	N = 64. T2D, adequate hepatic/renal function. Baseline HbA1c: 8.8%.	• HbA1c change: −1.9 percentage points (*p* < 0.0001) • Weight change: −4.8 kg (*p* < 0.0001) • Insulin stopped: 100% of users	Non-serious AEs (headache, tiredness) were transient. No serious events reported
Thamotharan et al. [24]	Case Studies 15-day illustration	N = 15. Elderly T2D patients with comorbidities.	• TIR: Improved to 86–97% from baseline of 3–75% • Insulin reduction: 14–29%	Not reported

**Table 8:** Interoperability, integration, and implementation barriers per study.

Authors	Interoperability Standard/Method	Level of EHR Integration	Reported Barriers/Challenges
Shamanna et al. [13,14,18]	IoT integration with commercial devices (Fitbit, scales) via Bluetooth/cellular network; proprietary platform.	Not reported.	Patient adherence to device usage and data logging.
Joshi, Shamanna et al. [16,19]	IoT integration; proprietary ML processing on a central platform.	Not reported.	(Implied) Complexity of managing multiple data streams in an RCT context.
Sarani Rad et al. [1]	Ontology aligned with HL7 FHIR standards.	Direct integration planned (EHRs are a key data source in the framework).	Data quality, privacy, security, and potential for biases.
Surian et al. [22]	Integration from multiple data sources, including EHR and clinical labs. Handles missing data without imputation.	High (integrates data directly from multiple EHR/lab systems).	Harmonizing data across different multi-ethnic cohorts.
Zhang et al. [2]	Knowledge graph (SPOKE) integrates 41 biomedical databases.	Future integration suggested via dashboards and LLMs.	Computational complexity of multi-omic data; scalability.
Thamotharan et al. [24]	Integration of multiple sensor types (BLE, NFC, WiFi) via IoT/IoMT devices.	Data from “patient records” used, implying some level of integration.	Real-time data processing at the edge; device discovery.
Cappon et al. [4]	Open-source software (MATLAB); compatible with standard CGM/pump data formats.	Not applicable.	Computational burden of the identification procedure.
Hughes et al. [21]	Interoperability with UVA/Padova simulator; accepts standard clinical data formats.	Not applicable.	Dependence of the “net-effect” signal on model inputs.

## Data Availability

Data are contained within the article.

## Abbreviations

The following abbreviations are used in this manuscript:

AI	Artificial Intelligence
AUC	Area Under Curve
AUC-ROC	Area Under Receiver Operating Characteristic Curve
BP	Blood Pressure
CDS	Clinical Decision Support
CGM	Continuous Glucose Monitoring
CKD	Chronic Kidney Disease
CVD	Cardiovascular Disease
DL	Deep Learning
DSS	Decision Support System
DT	Digital Twin
EHR	Electronic Health Record
FGM	Flash Glucose Monitoring
GDM	Gestational Diabetes Mellitus
GMF	Generalized Metabolic Fluxes
GV	Glycemic Variability
HbA1c	Hemoglobin A1c
HPC	High-Performance Computing
HTN	Hypertension
IoT	Internet of Things
JBI	Joanna Briggs Institute
LTI	Linear Time-Invariant
LSTM	Long Short-Term Memory
MAFLD	Metabolic Dysfunction-Associated Fatty Liver Disease
MARD	Mean Absolute Relative Difference
mHealth	Mobile Health
ML	Machine Learning
NAFLD	Non-Alcoholic Fatty Liver Disease
NN	Neural Networks
NPV	Negative Predictive Value
PPV	Positive Predictive Value
PRISMA-ScR	Preferred Reporting Items for Systematic Reviews and Meta-Analyses Extension for Scoping Reviews
PRO	Patient-Reported Outcomes
QoL	Quality of Life
RCT	Randomized Controlled Trial
RNN	Recurrent Neural Network
RWE	Real-World Evidence
SC	Standard Care
SMBG	Self-Monitoring of Blood Glucose
SOGMM	Subcutaneous Oral Glucose Minimal Model
SVM	Support Vector Machines
T1DM	Type 1 Diabetes Mellitus
T2DM	Type 2 Diabetes Mellitus
TAR	Time Above Range
TBR	Time Below Range
TIR	Time in Range
XAI	Explainable AI
